# Obese asthma phenotypes display distinct plasma biomarker profiles

**DOI:** 10.1002/clt2.12238

**Published:** 2023-03-22

**Authors:** Sophia Björkander, Susanna Klevebro, Natalia Hernandez‐Pacheco, Maura Kere, Sandra Ekström, Maria Sparreman Mikus, Marianne van Hage, Anna James, Inger Kull, Anna Bergström, Jenny Mjösberg, Christopher Andrew Tibbitt, Erik Melén

**Affiliations:** ^1^ Department of Clinical Science and Education Södersjukhuset Karolinska Institutet Stockholm Sweden; ^2^ Sachs' Children and Youth Hospital Södersjukhuset Stockholm Sweden; ^3^ CIBER de Enfermedades Respiratorias (CIBERES) Madrid Spain; ^4^ Institute of Environmental Medicine Karolinska Institutet Stockholm Sweden; ^5^ Centre for Occupational and Environmental Medicine, Region Stockholm Stockholm Sweden; ^6^ Department of Medicine, Solna Division of Immunology and Allergy Karolinska Institutet and Karolinska University Hospital Stockholm Sweden; ^7^ Department of Medicine Huddinge Centre for Infectious Medicine Karolinska Institutet Stockholm Sweden

**Keywords:** asthma, body mass index, inflammation, obesity, plasma biomarker

## Abstract

**Background:**

Obese asthma is a complex phenotype and further characterization of the pathophysiology is needed. This study aimed to explore inflammation‐related plasma biomarkers in lean and overweight/obese asthmatics.

**Methods:**

We elucidated levels of inflammation‐related plasma proteins in obese asthma phenotypes in the population‐based cohort BAMSE (Swedish: Children, Allergy, Milieu, Stockholm, Epidemiology) using data from 2069 24‐26‐year‐olds. Subjects were divided into lean asthma (*n* = 166), lean controls (*n* = 1440), overweight/obese asthma (*n* = 73) and overweight/obese controls (*n* = 390). Protein levels (*n* = 92) were analysed using the Olink Proseek Multiplex Inflammation panel.

**Results:**

Of the 92 included proteins, 41 were associated with lean and/or overweight/obese asthma. The majority of proteins associated with overweight/obese asthma also associated with overweight/obesity among non‐asthmatics. Beta‐nerve growth factor (BetaNGF), interleukin 10 (IL‐10), and matrix metalloproteinase 10 (MMP10) were associated only with lean asthma while C‐C motif chemokine 20 (CCL20), fibroblast growth factor 19 (FGF19), interleukin 5 (IL‐5), leukemia inhibitory factor (LIF), tumor necrosis factor ligand superfamily member 9 (TNFRSF9), and urokinase‐type plasminogen activator (uPA) were associated only with overweight/obese asthma. Overweight/obesity modified the association between asthma and 3 of the proteins: fibroblast growth factor 21 (FGF21), interleukin 4 (IL‐4), and urokinase‐type plasminogen activator (uPA). In the overweight/obese group, interleukin‐6 (IL‐6) was associated with non‐allergic asthma but not allergic asthma.

**Conclusion:**

These data indicate distinct plasma protein phenotypes in lean and overweight/obese asthmatics which, in turn, can impact upon therapeutic approaches.

## 1 INTRODUCTION

Asthma is a major non‐communicable disease related to reduced quality of life and high health‐care costs. The obese asthma syndrome is associated with female sex, more severe symptoms and poorer disease control compared to lean asthma. It is a complex phenotype related to both type‐2 and non‐type‐2 inflammation.^1^, ^2^ Adipose tissue is important in regulation of inflammation and obesity could affect the inflammation homeostasis.^3^ Changes in cytokine levels as well as altered immune responses have been suggested as potential mechanisms relating obesity to asthma.^1^, ^4^ Adipocytes and adipose tissue macrophages produce pro‐inflammatory cytokines such as IL‐6 which has been found in increased levels in asthmatics and has been related to low lung function.^5^, ^6^ Type 2 innate lymphoid cells have important functions in adipose tissue regulation of energy expenditure and metabolic homeostasis but are also related to asthma and asthma severity.^4^ Results from animal models suggest that systemic inflammation induced by obesity stimulate migration of innate lymphoid cells to the lungs where these cells could exhibit tissue dependant actions related to asthma.^7^ Adipose tissue has also been demonstrated in the outer wall of the large airways, where it correlated positively with BMI, wall thickness and granulocytes, highlighting a possible connection between obesity and asthma pathology.^8^

Biological drugs that target type‐2 pathways are of great interest in asthma treatment. However, targeting non‐type‐2 mechanisms in asthma patients is challenging and there is a need to identify novel, easily measurable biomarkers beyond classical type‐2 markers.^9^ Additionally, in obese asthmatics the predictive value of conventional biomarkers such as sputum eosinophils, serum eosinophils and fractional exhaled nitric oxide (FeNO) is poor.^10^ Research on immune cell phenotype and functionality is undoubtedly important to understand underlying mechanisms in asthma subtypes. Still, cell‐based assays are not feasible to routinely perform in clinical settings. There is a need to identify novel protein biomarkers in easily accessible tissues like blood plasma that could further inform us about involved pathways and disease mechanisms and help guide tailored treatment in asthma obesity phenotypes. The objective of this study was to explore plasma biomarkers related to lean and overweight/obese asthma in young adults. Since disease mechanisms and prevalence of overweight/obesity differ between allergic and non‐allergic asthma, association with biomarkers was also analysed in these sub‐phenotypes.

## 2 METHODS

The study population includes 2069 subjects born in 1994–1996 who completed a questionnaire and clinical examination at the 24‐year follow‐up of the ongoing population‐based Swedish cohort BAMSE (Barn/Child, Allergy, Milieu, Stockholm, Epidemiology).^11^

Individuals with asthma (*n* = 239) had a doctor's diagnosis ever of asthma in combination with symptoms of breathing difficulties and/or asthma medication use in the last 12 months. Individuals without asthma (*n* = 1830) are referred to as “controls”. All individuals were subdivided into “lean” (body mass index (BMI) < 25.0 kg/m^2^) or “overweight/obese” (BMI ≥ 25.0 kg/m^2^). Overweight/obese subjects with asthma were further subdivided into “allergic asthma” or “non‐allergic asthma” based on co‐incidental IgE‐sensitization (Figure S1).

### 2.1 Clinical variables

To assess IgE‐sensitization, sera were analysed for allergen‐specific IgE antibodies towards common airborne (birch, timothy, mugwort, house dust mite, cat, dog, horse, and mold) and food (egg, milk, cod, wheat, peanut, and soy) allergens by Phadiatop and fx5, respectively, using the ImmunoCAP System and a cut‐off of IgE ≥0.35 kU_A_/L (Thermo Fisher Scientific, Uppsala, Sweden).^12^

The Asthma Control Test (ACT) was used to assess the level of asthma control.^13^ An eosinophil blood concentration of ≥0.3 × 10^9^/L was used to define eosinophilic asthma according to the European Respiratory Society guidelines.^14^

Rhinitis was defined as symptoms from eye or nose because of furred animals or pollen (without having a cold) in the last 12 months prior to the questionnaire.^15^ Eczema was defined as any itchy skin rash in the last year in combination with 3 out of 4 following criteria: (i) dry skin in the last year, (ii) eczema onset <2 years of age, (iii) history of flexural eczema, (iv) history of asthma and/or rhinitis.^16^

Smoking and snuff use were defined as no, occasionally, and daily based on answers in the questionnaire. Weight and body fat percentage were measured using a Tanita MC 780 body composition monitor. FeNO measurements were performed using the Exhalyzer® D (EcoMedics Ltd) with the Air Safety Eco Slimline filter, cat No 4222/01 (Air Safety LTD), and the Spiroware 3.6.1 software. Lung function testing was performed through spirometry according to ERS/ATS criteria using the Jaeger spirometry apparatus and SentrySuite 2.17.^17^ The forced expiratory flow during 1 s (FEV_1_) and forced vital capacity (FVC) were not allowed to differ more than 150 mL or 5% from the previous value. The subjects received 4 × 0.1 mg Airomir inhalation (beta‐2 agonist) and re‐did the test after 15 min to investigate the degree of lung function reversibility. FEV1/FVC are presented as z‐scores using reference values from the Global Lung Initiative.^18^

### 2.2 Olink^TM^ multiplex protein assay

Venous blood was collected in EDTA tubes and plasma was obtained by centrifugation, aliquoted, and stored at −80° Celsius. Samples were collected during the clinical examination without specific requirements of prior fasting or time of sampling. The expression of 92 protein biomarkers in plasma was analysed by the Proseek Multiplex Inflammation Panel (Olink Biosciences, Uppsala, Sweden) as described in^19^. Data are expressed as normalized protein expression (NPX) on a log2 scale calculated from normalized Ct values. Protein abbreviations are used in tables throughout the manuscript (full names in Table S1).

### 2.3 Statistical analysis

The chi‐square, Fisher's exact, Mann‐Whitney U or Kruskal‐Wallis tests were used to analyse differences in baseline and clinical variables. The expression levels of inflammation‐related proteins in the lean asthma and overweight/obese groups were investigated by a crude or sex‐adjusted multinomial logistic regression model with the lean control group as the reference. To entangle if the association between plasma proteins and asthma differed depending on BMI status, an interaction term (protein*BMI‐group) was included in a binary logistic regression model with asthma as the outcome. To further explore the difference in biomarkers related to allergic and non‐allergic asthma a multinomial regression model with the overweight/obese control group as the reference was used to analyse these sub‐phenotypes. Sex, BMI (continuous) and body fat percentage were included as covariates in the adjusted model.

### 2.4 Ethics statement

The study was approved by the Regional Ethics Committee in Stockholm (DNR 2016/1380‐31/2) and conducted in accordance with the Declaration of Helsinki. All participants provided written informed consent.

## 3 RESULTS

Baseline and clinical variables are shown in Table 1. The study subjects were divided as described into lean asthma (*n* = 166), lean controls (*n* = 1440), overweight/obese asthma (*n* = 73) and overweight/obese controls (*n* = 390). Subjects with asthma were more often female (62%), IgE‐sensitized to airborne and food allergens, had rhinitis and eczema, experienced more respiratory infections, had higher BMI, body fat percentage, FeNO, and poorer lung function (Table 1).

**TABLE 1:** Baseline and clinical variables for the main study population.

		LEAN (BMI < 25.0)					OVERWEIGHT/OBESE (BMI ≥ 25.0)					
		Control (*n* = 1440)		Asthma (=166)		*p*	Control (*n* = 390)		Asthma (*n* = 73)		*p*	*p* overall
		*n*	%	*n*	%	Fisher's/Chi2	*n*	%	*n*	%	Fisher's/Chi2	Chi2
Sex	Female	816	56.7	106	63.9	0.082	181	46.4	42	57.5	0.097	**<0.001**
Eosinophilic asthma^a^	Yes			34	20.5				18	25.0		ns^b^
Sensitization, any	Yes	560	38.9	127	76.5	**<0.001**	176	45.1	52	71.2	**<0.001**	**<0.001**
Sensitization to airborne allergens	Yes	542	37.6	125	75.3	**<0.001**	172	44.5	51	69.9	**<0.001**	**<0.001**
Sensitization to food allergens	Yes	82	5.7	49	29.5	**<0.001**	25	6.4	20	27.4	**<0.001**	**<0.001**
Rhinitis	Yes	382	26.7	111	66.9	**<0.001**	108	28.4	45	62.5	**<0.001**	**<0.001**
Eczema	Yes	214	14.9	66	39.8	**<0.001**	70	18.1	28	38.4	**<0.001**	**<0.001**
Respiratory infections^c^	Never	183	12.8	14	8.5	**<0.001**	50	13.0	7	9.7	**0.033**	**<0.001**
	1–3 times	944	66.1	86	52.1		251	65.2	39	54.2		
	≥4 times	183	21.1	65	39.4		84	21.8	26	36.1		
Smoking	No	1157	80.4	127	76.5	ns	297	76.2	61	83.6	ns	**0.001**
	Occasionally	197	13.7	24	14.5		43	11.0	7	9.6		
	Daily	85	5.9	15	9.0		50	12.8	5	6.9		
Snuff	No	1251	86.9	145	87.4	ns	326	83.6	64	87.7	ns	ns
	Occasionally	63	4.4	6	3.6		18	4.6	4	5.5		
	Daily	126	8.8	15	9.0		46	11.8	5	6.9		

		Median	Range	Median	Range	Mann‐Whitney	Median	Range	Median	Range	Mann‐Whitney	Kruskal‐Wallis
Asthma control test				23.0	11.0–25.0				23.0	16.0–25.0		ns^d^
BMI		21.7	15.1–25.0	22.0	16.6–25.0	0.062	27.1	25.0–47.4	28.6	25.0–56.3	**<0.001**	**<0.001**
Body fat percentage		20.0	3.4–38.9	22.4	6.0–36.7	**0.010**	29.2	10.3–47.3	32.0	15.1–47.0	**0.003**	**<0.001**
Blood eosinophil conc.		0.1	0.0–2.5	0.2	0.0–1.2	**<0.001**	0.1	0.0–0.7	0.1	0.0–1.3	**0.039**	**<0.001**
Blood neutrophil conc.		3.3	0.8–14.4	3.3	1.5–10.6	ns	3.8	1.2–10.3	3.8	1.2–7.4	ns	**<0.001**
FeNO		12.0	5.0–180.0	15.0	5.0–124.0	**<0.001**	11.0	5.0–125.0	14.0	5.0–133.0	**0.021**	**<0.001**
Spirometry z‐scores	pre‐Fev1/FVC	−0.3	−3.1–2.8	−0.6	−2.7–1.8	**<0.001**	−0.5	−2.7–1.5	−0.8	−2.8–1.3	**0.009**	**<0.001**
	post‐Fev1/FVC	0.2	−2.1–2.7	0.0	−2.5–2.2	**0.003**	−0.1	−2.3–1.8	−0.3	−2.0–1.2	**0.003**	**<0.001**

*Note*: *p*‐values <0.05 indicated in bold.

Abbreviations: BMI, Body mass index; conc., concentration; FeNO, fractional exhaled nitric oxide; ns, non‐significant.

^a^ Defined as a blood eosinophil concentration of ≥0.3 × 10^9^/L in combination with asthma diagnosis.

^b^ Fisher´s exact test comparing groups with asthma.

^c^ Number of self‐reported respiratory infections (cold with rhinitis, coughing, fever) over the last 12 months.

^d^ Mann‐Whitney *U*‐test comparing groups with asthma.

### 3.1 Association with biomarkers in lean and overweight/obese asthma

In the multinomial regression, levels of 41 proteins were associated with either lean or overweight/obese asthma, of which Beta‐nerve growth factor (BetaNGF), interleukin 10 (IL‐10), and matrix metalloproteinase 10 (MMP10) were associated only with lean asthma while C‐C motif chemokine 20 (CCL20), fibroblast growth factor 19 (FGF19), interleukin 5 (IL‐5), leukemia inhibitory factor (LIF), tumor necrosis factor ligand superfamily member 9 (TNFRSF9), and urokinase‐type plasminogen activator (uPA) were associated only with the overweight/obese asthma phenotype. The remaining 32 proteins also associated with the overweight/obese control phenotype (Table 2).

**TABLE 2:** Multinomial logistic regression analysis to investigate associations of plasma proteins with lean and overweight/obese asthma.

	Lean asthma (*n* = 166)				Overweight/obese asthma (*n* = 73)				Overweight/obese control^a^ (*n* = 390)			
Protein	RRR (95% CI)	*p*	RRR (95% CI)^adj^	*p* ^adj^	RRR (95% CI)	*p*	RRR (95% CI) ^adj^	*p* ^adj^	RRR (95% CI)	*p*	RRR (95% CI)^adj^	*p* ^adj^
ADA	1.0 (0.7,1.5)	0.908	1.1 (0.8,1.7)	0.534	1.8 (1.1,3.0)	**0.029**	1.9 (1.1,3.1)	**0.021**	2.1 (1.6,2.7)	**<0.001**	2.0 (1.5,2.5)	**<0.001**
AXIN‐1	1.0 (0.9,1.2)	0.913	1.1 (0.9,1.3)	0.522	1.3 (1.0,1.7)	0.061	1.3 (1.0,1.7)	**0.045**	1.5 (1.3,1.7)	**<0.001**	1.4 (1.3,1.6)	**<0.001**
BetaNGF	1.9 (1.1,3.3)	**0.033**	1.9 (1.1,3.4)	**0.025**	0.6 (0.1,2.8)	0.486	0.6 (0.1,2.9)	0.494	0.9 (0.5,1.7)	0.830	0.9 (0.5,1.6)	0.653
CCL3	1.3 (0.9,1.9)	0.139	1.4 (1.0,2.0)	0.086	2.6 (1.8,3.9)	**<0.001**	2.6 (1.8,3.8)	**<0.001**	2.3 (1.8,2.9)	**<0.001**	2.2 (1.8,2.8)	**<0.001**
CCL4	1.1 (0.8,1.5)	0.621	1.1 (0.8,1.6)	0.378	1.9 (1.3,2.7)	**0.002**	1.9 (1.3,2.8)	**0.001**	1.9 (1.5,2.3)	**<0.001**	1.8 (1.5,2.2)	**<0.001**
CCL11	0.6 (0.4,1.0)	**0.034**	0.7 (0.5,1.1)	0.089	1.2 (0.7,2.0)	0.625	1.2 (0.7,2.1)	0.589	1.0 (0.7,1.3)	0.739	0.8 (0.6,1.1)	0.186
CCL19	1.2 (0.9,1.6)	0.122	1.2 (1.0,1.6)	0.118	1.9 (1.4,2.6)	**<0.001**	1.9 (1.4,2.6)	**<0.001**	1.4 (1.2,1.7)	**<0.001**	1.5 (1.2,1.7)	**<0.001**
CCL20	1.0 (0.9,1.3)	0.654	1.1 (0.9,1.3)	0.594	1.3 (1.0,1.7)	**0.031**	1.3 (1.0,1.7)	**0.031**	1.1 (1.0,1.2)	0.203	1.1 (0.9,1.2)	0.272
CD5	0.9 (0.6,1.3)	0.660	0.9 (0.6,1.4)	0.722	1.6 (1.0,2.6)	**0.037**	1.6 (1.0,2.6)	**0.036**	1.7 (1.3,2.1)	**<0.001**	1.7 (1.3,2.1)	**<0.001**
CD40	1.0 (0.7,1.5)	0.814	1.1 (0.8,1.6)	0.540	1.8 (1.1,3.1)	**0.029**	1.9 (1.1,3.2)	**0.023**	2.1 (1.6,2.7)	**<0.001**	2.0 (1.5,2.6)	**<0.001**
CDCP1	1.8 (1.1,2.8)	**0.012**	1.8 (1.1,2.8)	**0.014**	8.7 (5.4,13.9)	**<0.001**	8.7 (5.4,13.9)	**<0.001**	4.5 (3.3,6.1)	**<0.001**	4.6 (3.4,6.2)	**<0.001**
CSF1	2.0 (1.0,4.1)	0.055	1.8 (0.9,3.6)	0.126	3.7 (1.3,10.3)	**0.011**	4 (1.4,11.5)	**0.011**	2.3 (1.4,3.8)	**0.001**	3.2 (1.9,5.5)	**<0.001**
CST5	0.8 (0.5,1.2)	0.226	0.8 (0.5,1.2)	0.282	0.4 (0.2,0.8)	**0.009**	0.4 (0.2,0.8)	**0.010**	0.6 (0.4,0.8)	**<0.001**	0.5 (0.4,0.7)	**<0.001**
CXCL5	1.0 (0.9,1.2)	0.814	1.0 (0.9,1.2)	0.838	1.3 (1.0,1.7)	**0.039**	1.3 (1.0,1.7)	**0.040**	1.2 (1.1,1.4)	**0.001**	1.2 (1.1,1.4)	**0.001**
ENRAGE	1.0 (0.7,1.3)	0.774	1.0 (0.8,1.4)	0.772	1.6 (1.1,2.2)	**0.009**	1.6 (1.2,2.3)	**0.006**	1.5 (1.2,1.7)	**<0.001**	1.4 (1.1,1.6)	**0.001**
FGF19	1.0 (0.8,1.2)	0.916	1.0 (0.8,1.2)	0.931	0.7 (0.5,0.9)	**0.003**	0.7 (0.5,0.9)	**0.003**	0.9 (0.8,1.1)	0.325	0.9 (0.8,1.1)	0.341
FGF21	0.9 (0.8,1.1)	0.238	0.9 (0.8,1.1)	0.219	1.5 (1.3,1.8)	**<0.001**	1.5 (1.3,1.8)	**<0.001**	1.2 (1.1,1.4)	**<0.001**	1.2 (1.1,1.4)	**<0.001**
FGF23	1.0 (0.7,1.4)	0.906	0.9 (0.6,1.4)	0.780	1.7 (1.2,2.5)	**0.007**	1.7 (1.2,2.6)	**0.007**	1.5 (1.2,1.9)	**<0.001**	1.6 (1.3,2.0)	**<0.001**
HGF	0.9 (0.6,1.4)	0.808	1.1 (0.7,1.6)	0.747	4.7 (2.8,8.0)	**<0.001**	5.1 (3.0,8.7)	**<0.001**	3.4 (2.6,4.5)	**<0.001**	3.3 (2.4,4.3)	**<0.001**
IL‐5	1.0 (0.9,1.2)	0.976	1.0 (0.9,1.2)	0.974	1.3 (1.1,1.5)	**0.001**	1.3 (1.1,1.5)	**0.001**	1.0 (0.9,1.1)	0.641	1.0 (0.9,1.1)	0.550
IL‐6	0.9 (0.7,1.2)	0.569	0.9 (0.7,1.2)	0.533	2.9 (2.2,3.9)	**<0.001**	2.9 (2.2,4.0)	**<0.001**	2.2 (1.9,2.7)	**<0.001**	2.2 (1.9,2.7)	**<0.001**
IL‐7	1.3 (1.0,1.7)	0.059	1.3 (1.0,1.7)	**0.043**	1.6 (1.1,2.3)	**0.010**	1.6 (1.1,2.3)	**0.009**	1.5 (1.2,1.7)	**<0.001**	1.4 (1.2,1.7)	**<0.001**
IL‐10	1.3 (1.0,1.5)	**0.020**	1.3 (1.0,1.5)	**0.020**	1.1 (0.7,1.5)	0.710	1.1 (0.7,1.5)	0.707	1.0 (0.9,1.2)	0.775	1.0 (0.8,1.2)	0.883
IL‐10RB	1.5 (0.8,2.8)	0.228	1.5 (0.8,2.9)	0.210	5.5 (2.4,12.7)	**<0.001**	5.5 (2.4,12.7)	**<0.001**	3.0 (1.9,4.7)	**<0.001**	3.0 (1.9,4.6)	**<0.001**
IL‐12B	0.8 (0.6,1.1)	0.180	0.8 (0.6,1.1)	0.113	1.7 (1.1,2.6)	**0.015**	1.7 (1.1,2.6)	**0.015**	1.3 (1.1,1.6)	**0.007**	1.4 (1.1,1.7)	**0.001**
IL‐18	1.3 (0.9,1.8)	0.138	1.3 (1.0,1.8)	0.086	2.6 (1.7,4.0)	**<0.001**	2.6 (1.7,4.1)	**<0.001**	1.9 (1.5,2.4)	**<0.001**	1.8 (1.5,2.3)	**<0.001**
IL‐18R1	1.1 (0.7,1.8)	0.534	1.2 (0.8,1.8)	0.427	5.9 (3.2,11.1)	**<0.001**	6 (3.2,11.2)	**<0.001**	4.1 (3.0,5.7)	**<0.001**	4.0 (2.9,5.5)	**<0.001**
LAPTGFβ1	1.1 (0.7,1.6)	0.794	1.1 (0.7,1.7)	0.613	1.9 (1.1,3.2)	**0.024**	1.9 (1.1,3.3)	**0.022**	1.8 (1.4,2.3)	**<0.001**	1.7 (1.3,2.2)	**<0.001**
LIF	0.9 (0.6,1.3)	0.547	0.9 (0.6,1.3)	0.578	1.5 (1.1,2.0)	**0.007**	1.5 (1.1,1.9)	**0.008**	1.2 (0.9,1.4)	0.154	1.2 (0.9,1.4)	0.169
MCP1	0.8 (0.5,1.2)	0.274	0.9 (0.6,1.3)	0.457	2.3 (1.4,3.6)	**0.001**	2.3 (1.4,3.6)	**<0.001**	1.8 (1.4,2.3)	**<0.001**	1.7 (1.3,2.2)	**<0.001**
MCP3	1.4 (1.0,2.0)	0.058	1.4 (1.0,2.0)	**0.048**	3.2 (2.3,4.5)	**<0.001**	3.2 (2.3,4.5)	**<0.001**	2.3 (1.9,2.9)	**<0.001**	2.3 (1.9,2.9)	**<0.001**
MCP4	1.0 (0.8,1.2)	0.835	1.0 (0.8,1.3)	0.882	1.8 (1.3,2.4)	**0.001**	1.8 (1.3,2.5)	**<0.001**	1.5 (1.3,1.7)	**<0.001**	1.4 (1.2,1.7)	**<0.001**
MMP10	1.4 (1.1,1.8)	**0.002**	1.4 (1.1,1.8)	**0.002**	0.9 (0.6,1.3)	0.601	0.9 (0.6,1.3)	0.603	0.8 (0.7,1.0)	**0.036**	0.8 (0.7,1.0)	**0.030**
OSM	1.1 (0.9,1.2)	0.526	1.1 (0.9,1.3)	0.397	1.3 (1.0,1.6)	**0.021**	1.3 (1.0,1.7)	**0.020**	1.4 (1.2,1.6)	**<0.001**	1.4 (1.2,1.5)	**<0.001**
PDL1	1.0 (0.6,1.5)	0.876	1.1 (0.7,1.6)	0.762	1.7 (1.0,2.8)	0.056	1.7 (1.0,2.8)	**0.046**	1.7 (1.3,2.2)	**<0.001**	1.6 (1.2,2.0)	**0.002**
TNFRSF9	1.1 (0.7,1.8)	0.608	1.2 (0.8,2.0)	0.376	2.5 (1.3,5.0)	**0.008**	2.6 (1.3,5.2)	**0.007**	1.5 (1.0,2.0)	**0.025**	1.3 (0.9,1.8)	0.120
TNFSF14	1.1 (0.9,1.5)	0.352	1.2 (0.9,1.6)	0.179	1.8 (1.3,2.6)	**0.001**	1.9 (1.3,2.7)	**0.001**	1.9 (1.6,2.3)	**<0.001**	1.8 (1.5,2.2)	**<0.001**
TRAIL	1.2 (0.7,1.9)	0.562	1.4 (0.9,2.4)	0.178	4.0 (2.1,7.6)	**<0.001**	4.5 (2.3,8.8)	**<0.001**	3.7 (2.6,5.2)	**<0.001**	3.5 (2.4,5.0)	**<0.001**
TRANCE	0.9 (0.7,1.2)	0.402	1.0 (0.7,1.2)	0.723	1.8 (1.2,2.6)	**0.004**	1.9 (1.2,2.7)	**0.002**	2.0 (1.6,2.4)	**<0.001**	1.9 (1.6,2.3)	**<0.001**
uPA	1.2 (0.7,1.9)	0.513	1.3 (0.8,2.1)	0.312	2.7 (1.5,5.1)	**0.001**	2.8 (1.5,5.2)	**0.001**	1.0 (0.7,1.4)	0.941	0.9 (0.6,1.2)	0.398
VEGFA	1.4 (0.9,2.4)	0.164	1.5 (0.9,2.5)	0.158	5.9 (3.1,11.4)	**<0.001**	5.9 (3.1,11.4)	**<0.001**	4.2 (3.0,6.0)	**<0.001**	4.2 (3.0,5.9)	**<0.001**

*Note*: Reference: lean controls (*n* = 1440). *p*‐values <0.05 indicated in bold.

^a^ Proteins that show significant associations only in the overweight/obese control group are not displayed.

^adj^ adjusted for sex.

### 3.2 Effect modification of overweight/obesity on the association between biomarker and asthma

**FIGURE 1:**
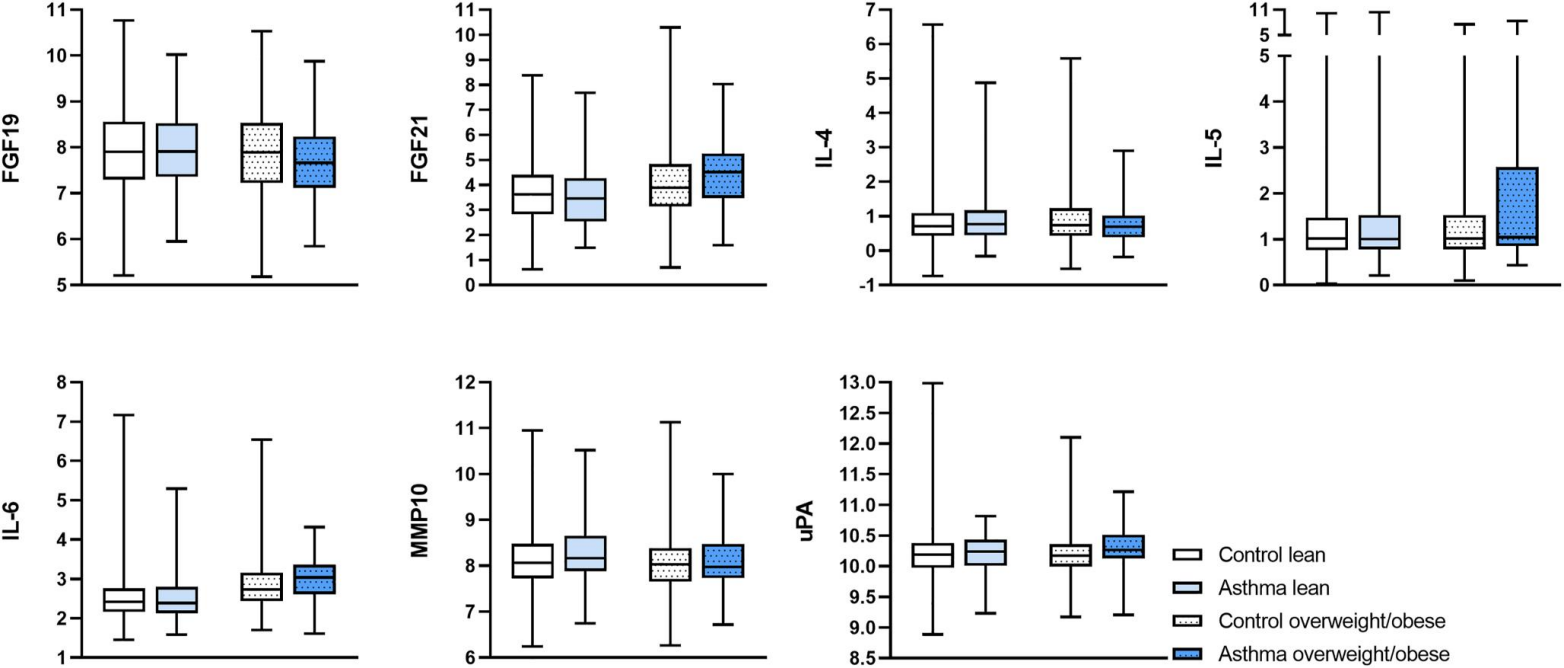
Boxplots of proteins with significant effect modification of overweight/obesity on the association of plasma proteins with asthma. Levels of FGF‐19, FGF‐21, IL‐4, IL‐5, IL‐6, MMP‐10, and uPA are expressed as normalized protein expression units. The box and whiskers cover minimum to maximum values with the central line as median.

We identified 14 proteins associated with asthma in the lean and/or overweight/obese BMI‐groups in the logistic model of asthma. Effect modification by overweight/obesity was significant in 3 of the proteins in the sex‐adjusted model: fibroblast growth factor 21 (FGF21), interleukin 4 (IL‐4), and uPA (Table 3, Figure 1).

**TABLE 3:** Logistic regression analysis to investigate effect modification of overweight/obesity on the associations of plasma proteins with asthma.

	Odds ratio of asthma ‐ lean				Odds ratio of asthma – overweight/obese					
Protein	Or (95% CI)	*p*	Or (95% CI)^adj^	p^adj^	Or (95% CI)	*p*	Or (95% CI)^adj^	p^adj^	*p*_interact^a^	*p*_interact^a^ ^adj^
BetaNGF	1.9 (1.1,3.4)	**0.032**	2.0 (1.1,3.5)	**0.024**	0.6 (0.1,3.2)	0.590	0.7 (0.2,3.4)	0.681	0.213	0.233
CCL11	0.6 (0.4,0.9)	**0.023**	0.7 (0.5,1.0)	0.083	1.2 (0.7,2.3)	0.494	1.4 (0.7,2.6)	0.313	0.074	0.070
CDCP1	1.7 (1.1,2.6)	**0.013**	1.7 (1.1,2.6)	**0.018**	2.1 (1.3,3.3)	**0.003**	2.0 (1.3,3.3)	**0.003**	0.579	0.551
FGF19	1.0 (0.8,1.2)	0.956	1.0 (0.8,1.2)	0.996	0.7 (0.5,1.0)	**0.021**	0.7 (0.6,1.0)	**0.027**	**0.047**	0.061
FGF21	0.9 (0.8,1.1)	0.240	0.9 (0.8,1.1)	0.220	1.2 (1.0,1.5)	**0.035**	1.2 (1.0,1.4)	**0.042**	**0.016**	**0.018**
IL‐4	1.1 (0.9,1.3)	0.535	1.1 (0.9,1.3)	0.485	0.7 (0.4,1.0)	**0.042**	0.7 (0.4,1.0)	0.061	**0.037**	**0.047**
IL‐5	1.0 (0.9,1.2)	0.981	1.0 (0.9,1.2)	0.994	1.3 (1.1,1.5)	**0.008**	1.3 (1.1,1.5)	**0.012**	**0.043**	0.052
IL‐6	0.9 (0.7,1.2)	0.622	0.9 (0.7,1.2)	0.648	1.5 (1.0,2.1)	**0.034**	1.4 (1.0,2.0)	0.058	**0.049**	0.077
IL‐7	1.3 (1.0,1.7)	**0.045**	1.3 (1.0,1.7)	**0.028**	1.1 (0.7,1.7)	0.639	1.1 (0.7,1.7)	0.603	0.517	0.474
IL‐10	1.2 (1.0,1.5)	**0.031**	1.2 (1.0,1.5)	**0.030**	1.1 (0.6,1.9)	0.753	1.1 (0.7,1.9)	0.645	0.675	0.776
MCP3	1.3 (0.9,1.7)	0.116	1.3 (1.0,1.7)	0.104	1.6 (1.1,2.5)	**0.023**	1.7 (1.1,2.6)	**0.017**	0.347	0.311
MMP10	1.4 (1.1,1.8)	**0.002**	1.4 (1.1,1.8)	**0.002**	1.1 (0.7,1.6)	0.647	1.1 (0.7,1.6)	0.671	0.241	0.223
OPG	1.5 (1.0,2.4)	0.070	1.4 (0.9,2.2)	0.151	2.2 (1.0,4.8)	**0.047**	2.0 (0.9,4.4)	0.082	0.431	0.443
uPA	1.1 (0.7,1.8)	0.590	1.3 (0.8,2.0)	0.315	3.5 (1.6,7.9)	**0.002**	3.8 (1.7,8.4)	**0.001**	**0.018**	**0.022**

*Note*: *p*‐values <0.05 indicated in bold.

Abbreviations: CI, confidence interval; OR, odds ratio.

^a^ Interaction term: protein*body mass index group.

^adj^ adjusted for sex.

### 3.3 Biomarkers related to allergic and non‐allergic asthma among overweight/obese study subjects

**FIGURE 2:**
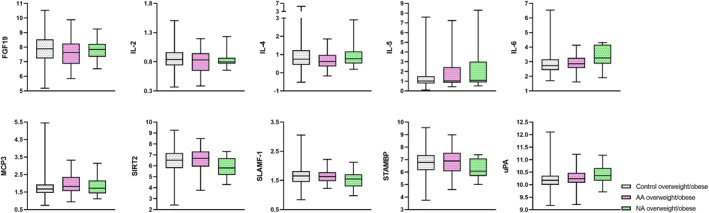
Boxplot of proteins with significant differences in expression levels between overweight/obese allergic asthma and non‐allergic asthma. Levels of FGF19, IL‐2, IL‐4, IL‐5, IL‐6, MCP3, SIRT2, SLAMF‐1, STAMBP and uPA are expressed as normalized protein expression units. The box and whiskers cover minimum to maximum values with the central line as median. AA: allergic asthma, NA: non‐allergic asthma.

Overweight/obese non‐allergic asthma (*n* = 21) was characterized by a higher proportion of females (81% compared to 48%, *p* = 0.009), lower prevalence of rhinitis (25% compared to 77%, *p* < 0.001) and eczema (19% compared to 46%, *p* < 0.001), and lower median FeNO (9 ppb compared to 20 ppb, *p* < 0.001) compared to overweight/obese allergic asthma (*n* = 52). Median BMI was 29.3 kg/m^2^ and 28.5 kg/m^2^ in the two groups (*p* = 0.300) and median body fat percentage was 36% in subjects with non‐allergic asthma compared to 31% in subjects with allergic asthma (*p* = 0.069). Differences in protein levels were examined in a multinomial logistic regression model with the overweight/obese control group as the reference. The proteins FGF19, interleukin 2 (IL‐2), IL‐4, and monocyte chemotactic protein 3 (MCP3) were associated with allergic asthma, whereas IL‐5, interleukin 6 (IL‐6), silent information regulator‐2‐like protein 2 (SIRT2), signalling lymphocytic activation molecule (SLAMF1), STAM‐binding protein (STAMBP), and uPA associated with non‐allergic asthma in the model adjusted for sex, BMI and body fat percentage (Table 4, Figure 2).

**TABLE 4:** Results from the multinomial regression of plasma proteins related to overweight/obese allergic and non‐allergic asthma.

	Overweight/obese allergic asthma (*n* = 52)				Overweight/obese non‐allergic asthma (*n* = 21)			
Protein	RRR (95% CI)	*p*	RRR (95% CI)^adj^	p^adj^	RRR (95% CI)	*p*	RRR (95% CI)^adj^	p^adj^
4‐EBP1	1.0 (0.7,1.3)	0.798	0.9 (0.6,1.3)	0.547	0.6 (0.3,0.9)	**0.022**	0.6 (0.3,1.0)	0.063
AXIN‐1	1.0 (0.7,1.4)	0.910	1.0 (0.7,1.4)	0.917	0.6 (0.4,0.9)	**0.025**	0.6 (0.4,1.1)	0.085
CASP8	1.1 (0.7,1.8)	0.665	1.1 (0.7,1.9)	0.665	0.3 (0.1,0.9)	**0.028**	0.4 (0.1,1.3)	0.142
CD244	1.4 (0.6,3.0)	0.418	1.3 (0.6,2.8)	0.503	0.3 (0.1,1.0)	**0.047**	0.3 (0.1,1.2)	0.086
CDCP1	1.8 (1.0,3.2)	**0.033**	1.5 (0.8,2.8)	0.240	2.7 (1.3,5.4)	**0.006**	1.8 (0.8,4.2)	0.170
CXCL6	1.0 (0.7,1.5)	0.924	1.0 (0.7,1.5)	0.942	0.5 (0.3,1.0)	**0.042**	0.5 (0.3,1.0)	0.067
FGF19	0.7 (0.5,0.9)	**0.012**	0.7 (0.5,0.9)	**0.016**	0.9 (0.5,1.4)	0.576	0.9 (0.6,1.5)	0.799
FGF23	0.7 (0.3,1.4)	0.307	0.5 (0.2,1.2)	0.106	2.2 (1.2,3.9)	**0.011**	1.8 (0.9,3.5)	0.081
Flt3L	1.0 (0.5,2.2)	0.971	0.9 (0.4,1.9)	0.709	3.1 (1.1,9.4)	**0.040**	1.8 (0.5,6.2)	0.327
IL‐2	0.2 (0.0,1.0)	**0.049**	0.2 (0.0,0.9)	**0.032**	0.8 (0.1,8.1)	0.819	0.6 (0.1,7.1)	0.693
IL‐4	0.5 (0.3,0.9)	**0.013**	0.5 (0.3,0.8)	**0.009**	1.0 (0.6,1.7)	0.968	1.1 (0.6,2.0)	0.638
IL‐5	1.2 (0.9,1.5)	0.136	1.2 (0.9,1.5)	0.140	1.5 (1.1,1.9)	**0.003**	1.5 (1.1,2.0)	**0.005**
IL‐6	1.1 (0.7,1.8)	0.657	0.8 (0.5,1.5)	0.550	2.4 (1.4,4.1)	**0.001**	2.1 (1.1,3.7)	**0.018**
IL‐10RB	1.5 (0.5,4.8)	0.485	1.1 (0.3,3.8)	0.826	4.9 (0.9,26.3)	0.067	5.5 (0.8,37.6)	0.083
MCP3	1.8 (1.1,2.9)	**0.011**	1.7 (1.0,2.7)	**0.043**	1.2 (0.5,2.7)	0.736	0.7 (0.2,1.9)	0.454
SIRT2	1.0 (0.8,1.3)	0.970	1.0 (0.7,1.3)	0.835	0.6 (0.4,0.9)	**0.019**	0.6 (0.4,1.0)	**0.050**
SLAMF‐1	1.1 (0.4,3.1)	0.866	1.0 (0.3,2.9)	0.984	0.2 (0.0,0.7)	**0.021**	0.2 (0.0,0.8)	**0.030**
STAMBP	1.0 (0.7,1.4)	0.974	1.0 (0.7,1.3)	0.792	0.5 (0.3,0.8)	**0.008**	0.6 (0.3,1.0)	**0.036**
uPA	2.4 (0.9,6.3)	0.067	2.4 (0.9,6.2)	0.077	8.1 (2.3,28.8)	**0.001**	8.6 (2.3,32.1)	**0.001**

*Note*: Reference: overweight/obese controls (*n* = 390). *p*‐values <0.05 indicated in bold.

^adj^Model adjusted for sex, body mass index and body fat percentage.

## 4 DISCUSSION

In this study, investigating young adults from a population‐based cohort with mild/moderate asthma, several plasma proteins were related to a lean and/or overweight/obese asthma phenotype. IL‐6 was associated with overweight/obese non‐allergic asthma, but not lean asthma or overweight/obese allergic asthma. Since IL‐6 is strongly associated with both sex and body composition measurements,^19^ it is important to note that our results remained significant after adjusting for sex, BMI, and body fat percentage. Levels of IL‐6 were reduced by a combined dietary and exercise intervention in a randomized trial.^20^ IL‐6 has also been linked to a severe asthma phenotype with worse lung function and more frequent exacerbations, independently of BMI.^5^ IL‐6 has a complex role in adipose tissue^21^ and is likely to have a complex role also in asthma pathogenesis that needs to be further elucidated.

FGF19 had a negative association with asthma among overweight/obese subjects whereas FGF21 had a positive association. FGF19 and FGF21 are known to be involved in energy homeostasis and obese subjects have lower levels of FGF19 and higher levels of FGF21.^22^ Our finding could indicate specific metabolic changes that are associated with asthma risk related to obesity. A recent study demonstrated that, in a model of severe steroid‐resistant asthma, inhibition of the FGF receptor prevented neutrophilic inflammation suggesting FGFs as potential therapeutic targets in asthma.^23^

uPA correlated with asthma among overweight/obese subjects and most notably in non‐allergic asthma. Reduced levels of the soluble uPA‐receptor (uPAR) have been shown 1 year after bariatric surgery^24^ as well as after a combined intervention of diet and exercise^25^ and uPAR also correlates with severe non‐allergic asthma and bronchial hyperresponsiveness.^26^, ^27^, ^28^ These results suggest importance of the uPA‐uPAR signalling pathway primarily in a non‐allergic asthma obesity phenotype.

Members of the matrix metalloproteinases have been linked to airway remodelling in several lung diseases and identified as potential therapeutic targets.^29^ In our study, MMP10 was one of few proteins associated only to lean asthma. We have recently shown that levels of MMP10 were associated with eosinophilic asthma.^30^ MMP10 has been related to airway remodelling and bronchial inflammation in asthma and regulates macrophage activity and subsequently the extent of inflammatory injury on the airways.^31^, ^32^

CUB domain‐containing protein 1 (CDCP1) has previously been suggested as a serum biomarker differentiating between poorly and well‐controlled asthma.^33^ In our study, CDCP1 associated with both lean and overweight/obese asthma and with overweight/obese allergic and non‐allergic asthma, however not after adjusting for sex, BMI and body fat percentage. These results suggest a potential role in asthma, however further studies to elucidate the link to different asthma phenotypes are needed.

An additional complexity of the obesity asthma phenotype is the co‐existence of other morbidities, such as obstructive sleep apnoea (OSA), which could potentially modify airway inflammation.^34^ Elevated levels of IL‐6 have been shown in the airways of patients with OSA^35^ and elevated levels of MMP9 were found in sputum of difficult‐to‐treat asthmatics with OAS.^36^

Strengths of our study include the well‐characterized cohort of young adults and the robustness of the Olink assay. Limitations are the inclusion of few subjects with severe asthma and non‐feasibility to analyse obese asthma separately from overweight asthma. Additionally, the cross‐sectional design of this study prevents conclusions regarding causation. The biomarkers identified in our results need to be further studied in interventional studies aimed at specific obesity asthma phenotypes.

In conclusion, this study highlights the importance of considering overweight/obesity as well as type‐2 and non‐type‐2 mechanisms when identifying potential new therapeutic targets in asthma treatment.

## AUTHOR CONTRIBUTIONS

**Sophia Bjorkander**: Conceptualization (equal); Formal analysis (lead); Methodology (equal); Visualization (lead); Writing—original draft (lead); Writing—review & editing (equal). **Susanna Klevebro**: Conceptualization (equal); Formal analysis (lead); Methodology (equal); Writing—original draft (lead); Writing—review & editing (lead). **Natalia Hernandez‐Pacheco**: Conceptualization (equal); Methodology (equal); Writing—review & editing (equal). **Maura Kere**: Conceptualization (equal); Visualization (equal); Writing—review & editing (equal). **Sandra Ekstrom**: Conceptualization (equal); Data curation (equal); Project administration (equal); Writing—review & editing (equal). **Maria Sparreman Mikus**: Writing—review & editing (equal). **Marianne van Hage**: Funding acquisition (supporting); Investigation (supporting); Writing—review & editing (equal). **Anna James**: Writing—review & editing (equal). **Inger Kull**: Data curation (equal); Funding acquisition (supporting); Investigation (equal); Project administration (equal); Resources (supporting); Writing—review & editing (equal). **Anna Bergstrom**: Data curation (equal); Funding acquisition (supporting); Investigation (equal); Project administration (equal); Resources (supporting); Writing—review & editing (equal). **Jenny Mjosberg**: Conceptualization (equal); Investigation (equal); Writing—review & editing (equal). **Christopher Andrew Tibbitt**: Conceptualization (equal); Investigation (equal); Writing—review & editing (equal). **Erik Melen**: Conceptualization (equal); Data curation (equal); Funding acquisition (lead); Investigation (equal); Methodology (equal); Project administration (equal); Resources (lead); Supervision (lead); Writing—original draft (supporting); Writing—review & editing (equal).

## CONFLICT OF INTEREST STATEMENT

EM reports lecture, consulting or advisory boards fees from AstraZeneca, Chiesi, Novartis and Sanofi outside the submitted work. SK reports lecture or advisory boards fees from Novartis and AstraZeneca outside the submitted work. MvH has received lecture fee from Thermo Fisher Scientific outside the submitted work. The other authors declare no conflicts of interest.

## Supporting information

Supporting Information S1

Supporting Information S2

## Data Availability

The data that support the findings of this study are available from the corresponding author upon reasonable request.
