# Supplementary figures and images for: Obese asthma phenotypes display distinct plasma biomarker profiles
Source: Clin Transl Allergy. 2023 Mar 22;13(3):e12238. doi: 10.1002/clt2.12238 (PMC10032201; doi:10.1002/clt2.12238)

**FIGURE S1: Inclusion of study subjects and groupings**

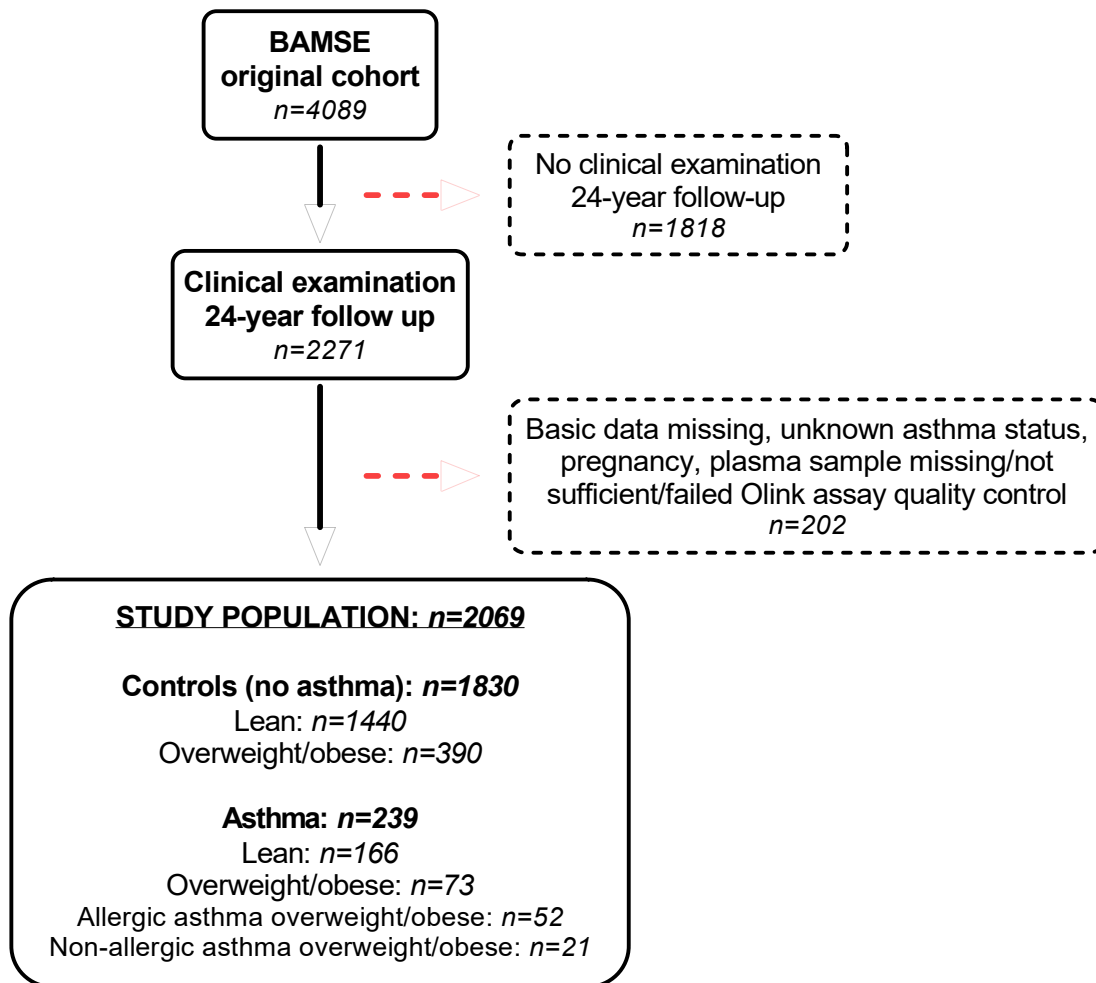

Supplement: Supplementary file 1 — Supporting Information S1 [file CLT2-13-e12238-s001.pdf]
