# Supplementary material for: Obese asthma phenotypes display distinct plasma biomarker profiles
Source: Clin Transl Allergy. 2023 Mar 22;13(3):e12238. doi: 10.1002/clt2.12238 (PMC10032201; doi:10.1002/clt2.12238)
Supplement: Supplementary file 2 — Supporting Information S2 [file CLT2-13-e12238-s002.pdf]

TABLE S1. Abbreviations and full names of all proteins in the manuscript.

Protein abbreviation	Protein full name	Protein abbreviation cont.	Protein full name cont.
4-EBP1	Eukaryotic translation initiation factor 4E-binding protein 1	LAPTGFβ1	Latency-associated peptide transforming growth factor beta 1
ADA	Adenosine Deaminase	LIF	Leukemia inhibitory factor
AXIN-1	Axin-1	MCP1	Monocyte chemotactic protein 1
BetaNGF	Beta-nerve growth factor	MCP3	Monocyte chemotactic protein 3
CASP8	Caspase 8	MCP4	Monocyte chemotactic protein 4
CCL3	C-C motif chemokine 3	MMP10	Matrix metalloproteinase 10
CCL4	C-C motif chemokine 4	OPG	Osteoprotegerin
CCL11	C-C motif chemokine 11	OSM	Oncostatin-M
CCL19	C-C motif chemokine 19	PDL1	Programmed cell death ligand 1
CCL20	C-C motif chemokine 20	SIRT2	SIR2-like protein 2
CD5	Cluster of Differentiation 5	SLAMF-1	Signaling lymphocytic activation molecule
CD40	Cluster of Differentiation 40	STAMBP	STAM-binding protein
CD244	Cluster of Differentiation 244	TNFRSF9	Tumor necrosis factor receptor superfamily member 9
CDCP1	CUB domain-containing protein 1	TNFSF14	Tumor necrosis factor ligand superfamily member 14
CSF1	Macrophage colony-stimulating factor 1	TRAIL	TNF-related apoptosis-inducing ligand
CST5	Cystatin D	TRANCE	TNF-related activation-induced cytokine
CXCL5	C-X-C motif chemokine 5	uPA	Urokinase-type plasminogen activator
CXCL6	C-X-C motif chemokine 6	VEGFA	Vascular endothelial growth factor A
ENRAGE	Protein S100-A12		
FGF19	Fibroblast growth factor 19		
FGF21	Fibroblast growth factor 21		
FGF23	Fibroblast growth factor 23		
Flt3L	Fms-related tyrosine kinase 3 ligand		
HGF	Hepatocyte growth factor		
IL-2	Interleukin 2		
IL-4	Interleukin 4		
IL-5	Interleukin 5		
IL-6	Interleukin 6		
IL-7	Interleukin 7		
IL-10	Interleukin 10		
IL-10RB	Interleukin 10 receptor beta		
IL-12B	Interleukin 12 subunit beta		
IL-18	Interleukin 18		
IL-18R1	Interleukin 18 receptor 1		
